# The relationship between the monitored performance of tutors and students at PBL tutorials and the marked hypotheses generated by students in a hybrid curriculum

**DOI:** 10.1080/10872981.2017.1270626

**Published:** 2017-01-06

**Authors:** Jonas I. Addae, Pradeep Sahu, Bidyadhar Sa

**Affiliations:** ^a^ Department of Preclinical Sciences, University of the West Indies, St. Augustine, Trinidad & Tobago; ^b^ Centre for Medical Sciences Education, Faculty of Medical Sciences, University of the West Indies, St. Augustine, Trinidad & Tobago

**Keywords:** Problem-based learning, monitoring, instrument, hypothesis quality, non-expert tutors, exploratory factor analysis

## Abstract

**Introduction**: There have been a number of published studies examining the link between the effectiveness of the problem-based learning (PBL) process and students’ performance in examinations. In a hybrid PBL/lectures curriculum, the results of such studies are of limited use because of the difficulty in dissociating the knowledge gained at lectures from that gained through PBL-related activities. Hence, the objectives of this study were: (1) to develop an instrument to measure the performance of tutors and students at PBL tutorials, and (2) to explore the contribution of such performances to the marks attained by students from the hypotheses generated at PBL tutorials.

**Methods**: A monitoring instrument for assessing the performances of non-expert tutors and students at tutorials was developed and validated using principal component analysis and reliability analysis. Also, a rubric was formulated to enable a content expert to assign marks to the quality of hypotheses generated.

**Results**: The monitoring instrument was found to be valid and reliable. There was a significant correlation between the performance of tutors at tutorials and hypotheses marks. In contrast, there was no significant correlation between the performance of students and hypotheses marks.

**Discussion**: The monitoring instrument is a useful tool for improving the PBL process, especially where the medical programme depends on non-expert PBL tutors. In addition to ensuring good PBL processes, it is important that students achieve the desired output at PBL tutorials by producing hypotheses that help them understand the basic sciences underlying the clinical cases. The latter is achieved by the use of an open-ended rubric by a subject expert to assign marks to the hypotheses, a method that also provides additional motivation to students to develop relevant and detailed hypotheses.

## Introduction

Problem-based learning (PBL) which has been a major part of medical education for half a century was promoted initially to improve application of knowledge by students to diagnose and manage clinical problems [1]. PBL, when properly applied, has been found to be an effective approach in medical education because it promotes constructive, self-directed, collaborative, and contextual learning [2,3]. However, there has been some debate on the superior effectiveness of PBL in the learning of basic medical knowledge and clinical skills [4,5]. Additionally, there is the tendency for cracks to develop and weaken the effectiveness of the PBL process and its outcome unless there is keen vigilance over the medical school’s PBL programme [6]. How students learn contributes significantly to what they learn [7]; hence, the focus of PBL should be on both the delivery process (how students learn) as well as the content (what students learn). Assessment, monitoring, and programme evaluation all contribute to improving what and how students learn [7]. A number of publications have reported the effectiveness of the PBL process by measuring students’ satisfaction of the process from surveys [8,9], or the knowledge acquired by students as measured by examinations [10–12]. However, there is hardly any reported work that uses an independent PBL specialist to monitor performance of tutor and students during PBL tutorial sessions and how their performance affects the quantity and quality of hypotheses generated by the tutorial group.

The medical school at the University of the West Indies, Trinidad and Tobago, uses a hybrid system of PBL and lectures/laboratory practicals. The school follows the seven-step systematic approach of PBL developed by the University of Linburg, Maastricht [1]. The medical school uses non-expert tutors with MD (or equivalent) or PhD backgrounds who would have some understanding of the content of the cases. A PBL group, which meets once a week, comprises 11–13 students and the tutor. A PBL session lasts approximately three hours and comprises two phases: (1) ‘problem-analysis phase’ during which students brainstorm a new problem, develop relevant hypotheses based on prior knowledge and generate learning objectives for self-study; (2) ‘reporting phase’ during which students discuss the objectives generated from the previous problem and revise the previous hypotheses. In the first two years of our medical programme, students are expected to focus their learning on identifying key issues in the problem and providing detailed explanations for the issues identified; they are not expected to provide diagnosis or a management plan for patients in the problem. The quantity and quality of relevant hypotheses generated by a tutorial group is marked by a content expert and the results given to the students before the next tutorial session.

The medical school recognizes the three key elements that are essential to a successful PBL programme: (1) the problem used to stimulate learning, (2) the tutors as facilitators of learning, and (3) the group work (or team work) that ensures interaction amongst the students [13]. The current study focused on the latter two elements with the following objectives: (1) to validate a monitoring instrument to measure the performance of tutors and students in a tutorial, i.e., how students learn; (2) to determine the extent to which the performance of tutors and students in a tutorial influenced the hypotheses generated by students, i.e., what students learn.

## Methods

### Developing a monitoring instrument

The study was conducted in accordance with the guidelines of the university’s ethics committee. The first part of the study was to validate a suitable monitoring instrument and determine its reliability. The items on the monitoring instrument were determined by medical education experts using information from published articles on the expected roles of tutor and students; the latter included the group leader, record keeper, and other students in the group [14–21]. Following feedback from experienced PBL tutors, the medical education experts settled on two constructs with 21 items: 11 items for evaluating the performance of students during PBL tutorial sessions and 10 items for evaluating the performance of students during the tutorials. The two constructs and items in the instrument are listed in Table 1. In order to test further the validity of the instrument, it was given to 40 randomly-chosen PBL tutors to rate the extent to which each item was appropriate for the construct being examined. The participants were asked to evaluate the items using a 5-point Likert scale: 1: strongly disagree, 2: disagree, 3: undecided, 4: agree, 5: strongly agree. The data obtained was subjected to an exploratory factor analysis (principal component analysis) with oblique (direct oblimin) rotation; the latter was chosen with the assumption that the factors would not be independent. The internal consistency of the items in each construct was determined by measuring Cronbach’s reliability coefficients.

**Table 1.** Summary of exploratory factor analysis of PBL monitoring instrument.

Items	Factor 1: Performance of students	Factor 2: Performance of tutor
Leader encourages all group members to participate in the discussion	**0.85**	0.06
Leader allows everyone to express his/her views	**0.82**	0.13
Students encourage cooperative behaviour in the group	**0.80**	0.43
Leader summarizes the views at appropriate period during the discussion	**0.72**	0.20
All students get involved in group discussions	**0.66**	0.36
Leader emphasizes clarification of different issues	**0.65**	0.03
Students respond to feedback from tutor	**0.63**	0.22
Record keeper participates in group discussions	**0.55**	0.46
Students ask stimulating questions	**0.55**	0.44
Leader starts PBL session on time	**0.50**	−0.08
Tutor encourages recording of contributions during brainstorming	0.16	**0.87**
Tutor encourages noting down hypotheses and learning objectives properly	0.11	**0.82**
Tutor creates a supportive and comfortable learning environment	−0.04	**0.75**
Tutor guides students in formulating learning objectives	0.45	**0.67**
Tutor promotes critical thinking skills	0.25	**0.67**
Tutor encourages cooperative behaviour in the group	0.12	**0.58**
Tutor encourages all students including less involved students to take part in discussion	0.26	**0.56**
Tutor ensures students adhere to polite norms of spoken communication	0.45	**0.56**
Tutor intervenes when discussion goes off track	0.54	**0.55**
Tutor ensures group follows the steps of PBL	0.44	**0.54**
		
Item not included		
Tutor facilitates self-directed learning	−0.01	0.44
		
Eigenvalues	7.01	3.22
Contribution to variance (%)	33.37	15.35
Cronbach’s alpha	0.87	0.86

Factor loadings of 0.50 or more are in bold.

### Monitoring performance of tutors & students and marking hypotheses generated by students

In the second part of the study, the instrument (with the same 5-point Likert scale as above) was used by an independent medical education specialist to monitor 23 PBL groups in years 1 and 2 (basic sciences) of the medical programme. The hypotheses generated by each PBL group for a particular clinical problem was marked by an independent content expert to generate a hypotheses mark for the group. The courses had five to eight clinical problems depending on the length of the course. Table 2 shows the rubric used to determine the hypotheses mark obtained by a group of students for a PBL problem, with the maximum mark being 10. The average hypotheses mark for the group was the mean of all the marks obtained during the course. Pearson’s product-moment correlation analysis was used to determine how the performance of tutors and students influenced the average hypotheses mark for the group. All statistical analyses were performed using SPSS (version 22) software.

**Table 2.** Rubric used to mark the hypotheses generated by PBL groups.

	A+ (10)	A (8)	B (7)	C (5)
**Critical issues**	All critical issues in the problem are identified	All critical issues in the problem are identified	Most of the critical issues in the problem are identified	Less than 50% of the critical issues in the problem are identified
**Explanations**	Detailed explanations are given for all critical issues. Issues not clearly stated in the problem are identified and properly explained	Detailed explanations are given for at least 75% of the issues identified	Detailed explanations are given for at least 50% of the issues identified	Detailed explanations are given for less than 50% of the issues identified

Numbers in brackets are the marks associated with the letter grades.

## Results

### Validation of monitoring instrument

Responses to the questionnaire were obtained from 31 tutors, giving a response rate of 78%. Table 1 shows the results of the principal component analysis of the 21 items with oblimin rotation. The Kaiser-Meyer-Olkin measure of sampling adequacy was found to be 0.60 which was above the recommended acceptable limit of 0.5 [22]. The scree plot showed an inflexion that justified retention of the two factors. Table 1 shows the eigenvalues and percentage of variance accounted for by each factor. Table 1 also shows the rotated factor loadings of all items with values of 0.5 or greater in bold. There were 10 items that clustered around factor 1, and another 10 items on factor 2. One item had a factor loading of less than 0.5 and was therefore excluded in determining the reliability of the instrument and in part 2 of the study.

A Cronbach’s alpha coefficient of 0.7 or more indicates a reliable scale [22,23]. The two factors, using the 10 items in each construct, had coefficients of approximately 0.87 and 0.86 (Table 1). None of the items in either factor had a ‘corrected item-total correlation’ of less than 0.4 or increased the alpha coefficient for the construct when the item was deleted. Hence, the reliability of the instrument was found to be acceptable and the items with bold rotated factors in Table 1 were used for part 2 of the study.

### Influence of performance of tutors and students in a tutorial on the hypotheses generated by students

In the second part of the study, the monitored performance of tutors and students in 23 PBL groups were compared to the hypotheses marks attained by the group of students. The mean score for students performance on the 5-point Likert scale was 3.7 ± 0.4 (mean ±s.d.), whilst that for tutor performance was 4.3 ± 0.4. The quality of hypotheses generated by all student groups which was marked over a maximum of 10 was 8.3 ± 0.8 (mean ±s.d.). Pearson’s product-moment correlation analysis showed a significant correlation between the monitored tutor performance and hypotheses mark attained by students (r = 0.44; p = 0.02, one-tailed). In contrast, the correlation coefficient between student performance and hypotheses mark was very low and not significant (r = 0.08; p = 0.35, one-tailed). Additionally, there was a significant correlation between the monitored tutor performance and students performance (r = 0.43, p = 0.02, one-tailed)

## Discussion

There have been several published assessment methods for PBL, many of which assess the process whilst others assess the outcome, e.g., knowledge content [8–11,24]. In order to improve the effectiveness of PBL as a learning tool, it is necessary to assess both the process and the outcome.

The instrument we developed for assessing the process initially had two constructs and 21 items. Following a factor analysis, one item was deleted because of low factor loading, thus the final instrument has 20 items, 10 of which clustered around students’ performance at PBL tutorials and the other 10 around tutors’ performance. The results of this part of the study indicate the instrument has both construct validity and internal consistency (reliability) and could be used to monitor the performances of tutors and students during PBL tutorials.

Schmidt (1983) proposed that information processing theory in educational psychology formed the bedrock of PBL, i.e., activation of prior knowledge, encoding specificity, and elaboration of knowledge. Additionally, cooperative learning (rather than competitive learning) is essential for the success of PBL [2,25]. Cooperative learning is promoted when students have shared goals and rewards, and optimize their complementary roles to achieve them. Another key advantage of PBL is to promote the development of clinical reasoning skills during the early stages of medical training. Familiarity with clinical cases leads to the formation of clearly defined illness scripts that enable clinicians (especially specialists) to quickly diagnose a clinical case. However, when confronted with unfamiliar cases (especially complex ones), doctors tend to unravel the case by using hypotheses generation in the form of self-explanations [26,27]. Hypotheses generation involves constructing linkages amongst items in the case and with the underlying mechanisms/reasons from biological, psychological, social, ethical, and legal perspectives. We promoted collaborative learning and the development of clinical reasoning skills in our programme by introducing a system in which the written hypotheses from PBL groups for each problem are marked by a content expert each week and the marks given back to the group, thus ensuring immediate feedback to students on their output. The marks attained by the group formed part of each student’s continuous assessment. This approach encouraged students not only to work together but also gave the faculty a sense of what students have learnt, and to address possible gaps in knowledge across the groups.

Some studies have found significant correlation between the score assigned to a student by a tutor and the student’s marks on traditional examinations, e.g., multiple choice questions; whilst others have found no such correlation [10–12]. The design of the current study differs from the other published ones in that (1) we examined the two separate constructs that contribute to the PBL process i.e. performances of students and tutors, (2) we reduced bias in scoring students and tutors at PBL tutorials by using the same medical education expert to do the scoring, (3) the marks obtained were directly associated with the PBL process because it was based solely on the hypotheses generated by the group at PBL and not on examination scores which are affected by other forms of learning in a hybrid medical education curriculum, and (4) the marks were attained by the whole group and not by individual students. Our study has added another dimension to the debate by the finding that the performance of tutors correlated significantly with the quantity and quality of hypotheses generated by students.

In the current study, the hypotheses marks achieved by students were not influenced by the variation in performance of students at tutorials. This suggests that the quantity and quality of hypotheses generated by students is dependent on factors other than the students’ performance in the PBL tutorial process. For example, the hypotheses generated would have reflected the depth of the students’ prior knowledge or the depth of the knowledge gained during the self-study step of the PBL process. This line of reasoning could be supported by the diverse learning resources online and technological tools that are currently available to medical students [28].

A number of studies have demonstrated that both expert and non-expert tutors do influence the PBL process, and the importance of having quality tutors for the PBL process [5,29,30]. In the current study, the performance of tutors had significant correlation with the performance of students during tutorials. In our programme, PBL tutors are trained to be effective facilitators by the medical school’s Centre for Medical Sciences Education. Hence, it was reassuring to note that tutor performance in this study was found to be significantly correlated with students’ performance during tutorial.

## Conclusion

An instrument has been developed to monitor the performance of tutors and students at PBL tutorials. The 20-item instrument with two constructs was shown to have good validity and reliability. Whilst having a good PBL process is essential, it is also important that the students’ achieve the desired output at PBL tutorials by producing hypotheses that are relevant to the clinical case being discussed. Hence, we have also provided a rubric for assessing the hypotheses generated by students during PBL tutorials. The results of this study emphasize the important role of the tutors in facilitating the performance of students at tutorials and getting them to engage in discussions that produce excellent hypotheses. The tutors, albeit non-expert, were found to be capable of influencing not only how students learn but also what they learn.
